# The draft genome sequence of *Kitasatospora* sp. HPM-01-4 isolated from Mentaos Pine Forest, heart of Borneo, Indonesia

**DOI:** 10.1128/mra.00739-25

**Published:** 2026-02-27

**Authors:** Dwi Ariyanti, Matin Nuhamunada, Nurul Izzati, Maya Fitriana, Aulia R. Widyaningrum, Immanuel Sanka, Ali B. Kusuma

**Affiliations:** 1 Biotechnology Department, Faculty of Life Sciences and Technology, Sumbawa University of Technology 563750 , Sumbawa, West Nusa Tenggara, Indonesia; 2 Research Collaboration Center for Thermophilic Enzyme, Jakarta, Indonesia; 3 Faculty of Biology, Universitas Gadjah Mada 59166, Yogyakarta, Indonesia; 4 Synthetic Biology Indonesia, Jakarta, Indonesia; 5 Indonesian Centre for Extremophile Bioresources and Biotechnology (ICEBB), Faculty of Life Sciences and Technology, Sumbawa University of Technology 563750 , Sumbawa, West Nusa Tenggara, Indonesia; California State University San Marcos, San Marcos, California, USA

**Keywords:** genomes, *Kitasatospora*, HPM-01-4, Borneo

## Abstract

The draft genome of *Kitasatospora* sp. HPM-01-4 was sequenced using the ONT PromethION platform, yielding 12 scaffolds with a total genome size of 9.5 Mb, comprising a 9.1 Mb main contig and 11 shorter contigs.

## ANNOUNCEMENT

We isolated *Kitasatospora* sp. HPM-01-4 in April 2024 from soil collected in the Mentaos Pine Forest, South Kalimantan, Indonesia (3.43467° S, 114.83342° E). Although genomes of *Kitasatospora* have been widely studied, genomic data for Indonesian isolates remain limited (1). Prior analyses of more than 40 *Kitasatospora* genomes reported an average of 34.1 ± 8.2 secondary metabolite biosynthetic gene clusters per genome (2), indicating substantial biosynthetic capacity within the genus. Therefore, whole-genome sequencing of HPM-01-4 may help characterize its biosynthetic potential. This bioactive strain is currently classified as *Kitasatospora* sp. (Actinomycetes). We collected 50 g of soil from 13 cm below the surface and transported it in a 50 mL Falcon tube in a cold box. Samples were air-dried for 7 days at room temperature in a sterile Petri dish. One gram of dried soil was serially diluted 1:10 and 1:1,000 in Ringer’s solution, then heat-treated at 55°C for 6 min. Aliquots of 20 μL of each dilution were spread on Actinomycetes Isolation Agar and incubated at 30°C for 7 days (3). A single colony obtained was streaked onto International Streptomyces Project 2 (ISP2) agar and incubated at 30°C for 7 days (4, 5). Biomass was collected from the plate culture and stored in 200 μL of 20 g/L glycerol stock at −20°C. Genomic DNA was extracted from 50 to 100 mg of cell pellets obtained from a 20 mL ISP2 broth culture incubated at 30°C with shaking at 150 rpm for 5 days. Genomic DNA was purified using the ZymoBIOMICS DNA Miniprep Kit according to the manufacturer’s protocol, and its concentration was measured with a Qubit 4 Fluorometer (Invitrogen), yielding 520 ng/μL. For the sequencing, 400 ng of genomic DNA was prepared using the ONT ligation sequencing gDNA Native Barcoding Kit (SQK-NBD114.24; Oxford Nanopore Technologies, UK) following protocol NBR_9169_v114_revI_15Sep2022 (with no size selection performed for ONT library preparation) and sequenced on a PromethION instrument using an FLO-PRO114M R10.4.1 flow cell. Basecalling was performed with Dorado v0.9.0 using the model dna_r10.4.1_e8.2_400bps_sup@v5.0.0. ONT sequencing generated 2.7 Gbp across 3.36 M raw reads, with an N50 read length of 1.8 Kbp. Reads were assembled and annotated using the EPI2ME wf-bacterial-genomics workflow. Assembly was performed with Flye v2.9.6 (6) and polished with Medaka v2.0.1 (7). Gene prediction and annotation were performed with Prokka v1.13.4 (8). Phylogenomic placement was conducted using GTDB-Tk v2.4.0 (9) with GTDB release 220 (10), which assigned the isolate to *Kitasatospora* sp. The assembly comprised 12 contigs covering 9.46 Mbp in length, with an N50 of 9.14 Mbp and a mean depth of ~289×. Genome completeness was 98.48% as assessed using CheckM analysis (11). The genome matches with *Kitasatospora cystarginea* (GCF_039533515.1) with 90.56% average nucleotide identity. From the annotation, we identified 7,811 protein-coding genes, 72 tRNAs, and 33 rRNA genes. The overall GC content from all contigs was 72.79%. Genome mining using antiSMASH v7.1.0 (12) revealed 51 biosynthetic gene cluster regions on the main chromosome, including 11 PKS-like (polyketide synthase) and 16 non-ribosomal peptide synthetase-like regions.

## Data Availability

All data are available under BioProject PRJNA1277368 and BioSample SAMN49107652: KS HPM 01 *Actinomycetota* (TaxID 1760). The raw reads have been deposited at SRA (accession number SRR33996622). Prokka and antiSMASH annotations are available on Zenodo (https://doi.org/10.5281/zenodo.17532396). The genome assembly information is available from NCBI (accession number GCA_051374625.1).
